# Life satisfaction, positive affect, depression and anxiety symptoms, and their relationship with sociodemographic, psychosocial, and clinical variables in a general elderly population sample from Chile

**DOI:** 10.3389/fpsyt.2023.1203590

**Published:** 2023-06-27

**Authors:** Sandra Saldivia, Joseph Aslan, Félix Cova, Claudio Bustos, Carolina Inostroza, Anabel Castillo-Carreño

**Affiliations:** ^1^Department of Psychiatry and Mental Health, Faculty of Medicine, Universidad de Concepción, Concepción, Chile; ^2^Doctoral Program in Psychology, Universidad de Concepción, Concepción, Chile; ^3^Department of Psychology, Faculty of Social Sciences, Universidad de Concepción, Concepción, Chile; ^4^Department of Fundamentals of Nursing and Public Health, Faculty of Nursing, Universidad de Concepción, Concepción, Chile

**Keywords:** elderly people, depression symptoms, anxiety symptoms, life satisfaction, positive affect, dominance analysis

## Abstract

**Background:**

This study aims to describe the relationship between life satisfaction, positive affect, depression and anxiety symptoms with sociodemographic, psychosocial and clinical variables, and to identify the relative importance of these predictor groups.

**Methods:**

We evaluated life satisfaction (SWLS), positive affect (PANAS), depressive (PHQ-9), and anxiety (GAI) symptoms and their association with sociodemographic, psychosocial and clinical variables in a multistage, random general population sample of fully functioning individuals aged 60–80 years from the Concepción province and Gran Santiago, Chile (*n* = 396). We performed weighted multiple regression analysis, considering the complex sample structure with age group, sex, and geographical area, complemented with general and conditional dominance analyses to estimate the relevance of the predictor groups.

**Results:**

We found significant associations with the geographical area, sex, age, education level, household members, having a partner, employment status, caregiver status, economic satisfaction, presence of chronic diseases, medication use, and alcohol use. Satisfaction with health was the most important predictor for positive affect (*p* < 0.001), depressive (*p* < 0.001), and anxiety (*p* < 0.001) symptoms, while alcohol use was the most significant predictor for life satisfaction (*p* < 0.001).

**Conclusion:**

Simultaneously studying the positive and negative dimensions of wellbeing and mental health in older adults allows for a more comprehensive perspective on the challenges faced during this stage of life. This study accounts for previously unknown associations and contributes to the identification of common and specific predictors in both dimensions.

## 1. Introduction

Life expectancy is increasing worldwide, and Chile is one of the Latin American countries with a high proportion of elderly people, reaching up to 11.4% of the total population in 2017 (1). This number is projected to increase to 32% by 2050 (2), posing a challenge to healthcare systems, particularly those entrusted with maintaining their wellbeing and mental health.

The comprehensive model of mental health, also known as the dual-continuum model, identifies two correlated yet distinct dimensions: one is the positive wellbeing or mental health, on the other hand are the mental health problems and disorders. Both dimensions have independent predictive value and should be considered when studying and assessing individuals’ mental health (3, 4). In this study, we focus on two of the most relevant components of positive wellbeing or mental health: life satisfaction and positive affect, as well as two of the most common mental health problems, depressive and anxious symptoms. Despite the increasing research on mental health problems in the elderly and their associated variables, studies on wellbeing and mental health problems in this population are still scarce.

Life satisfaction is the subjective perception of wellbeing or happiness in one’s life. It involves a comprehensive cognitive evaluation of one’s life in relation to their goals, expectations, and interests, which is influenced by the cultural context in which one inhabits (5). Higher life satisfaction is associated with various positive health outcomes, including a reduced risk of physical health problems and mortality, as well as better health behaviors (5). A recent systematic review exploring factors affecting life satisfaction in older adults identified thirteen factors that positively affect this perception (6). These factors include cognitive ability, daily life decisions, economic status, education level, health status, insurance, marital status, preferred living arrangement, quality and duration of sleep, religiosity, social functioning, social support, and housing satisfaction. The review also identified six factors negatively correlated with life satisfaction: physical limitations, childlessness, depression, discordant living arrangement, perceived discrimination, and aging (6). In a nationally representative sample of elderly individuals from Spain, a lower level of disability and retirement, as opposed to being employed, were predictive of higher life satisfaction. Conversely, a lower income level, depression, feelings of loneliness, a higher disability, and unemployment, compared to being employed, were associated with lower life satisfaction (7).

Positive affect (PA) refers to the experience of pleasurable emotions such as happiness, joy, excitement, enthusiasm, calmness, and contentment (8). In elderly people, PA is associated with longer life, minor physical decline and frailty, improved cardiovascular health, and more positive outcomes in chronic diseases (9). Conversely, depressive symptoms, loneliness, and low physical activity predict decreased PA among adults (7). A meta-analysis exploring the relationship of PA with mortality risk in older adults found that higher PA is associated with lower mortality risk, even after controlling for medical, psychological, and social factors (10).

Depression and anxiety commonly coexist in old age and are among the most prevalent mental disorders in the geriatric population (11). These disorders negatively affect health, and they are associated with disability, worsening of chronic conditions, increased suicide rates, as well as poorer quality of life (12). Furthermore, an increased depressive symptomatology is associated with decreased quality of life (13). Being female is associated with a higher prevalence of depressive and anxiety disorders (14), and lacking a partner and having a weak social support are associated with depressive symptoms (15). A recent systematic review and meta-analysis examining the prevalence and risk factors associated with depression, anxiety, and insomnia among different populations, including older adults, during the COVID-19 pandemic, found that factors associated with increased prevalence of depression and anxiety were: being female, having physical disorders, psychiatric disorders, alcohol use, COVID-19 infection, colleagues or family members infected, close contact with infected patients, high exposure risk, quarantine experience, and high concern about epidemics (16). The same study found that frequent exercise, high income, and good social support were associated with a lower risk of both depression and anxiety.

Long-term moderate or severe depressive symptoms significantly disrupt daily life, work, and family activities (17). They also lead to heightened susceptibility to physical discomfort, increased social isolation, as well as decreased social relationships and wellbeing (11, 18). Conversely, the prevalence of depression is lower among older individuals with better emotional skills, greater autonomy, as well as relationships with others (19). A recent umbrella review of published meta-analyses and systematic review assessing risk factors for depression in the elderly found the following factors associated with a higher depression risk: sleep disturbances, slow gait speed, living arrangement, cognitive function, chronic diseases, subthreshold depression, aspirin use, hearing loss, alcohol use disorders and heavy drinking, metabolic syndrome, poor vision, frailty, vascular risk, abdominal obesity, sleep duration, and individuals aged 80 or above (20).

Anxiety is more prevalent in older individuals with physical health conditions and disabilities, such as heart disease, stroke, and depression (21), as well as cognitive impairment (22). It is also associated with reduced quality of life, increased healthcare services utilization, and polypharmacy (23). Research indicates a negative association between age and anxiety among the elderly individuals. Additionally, the likelihood of reporting an anxiety disorder is higher among separated, divorced, and widowed relative to their married counterparts. Social support plays a significant role in mediating the effect of marital status on anxiety disorder (24). Two reviews have explored risk factors for anxiety in the elderly, their results confirming the influence of self-perceived health and highlighting biological risk factors like hearing and vision loss, psychological risk factors related to personality traits (locus of control and neuroticism), dysfunctional coping strategies and psychopathology, and social risk factors such as lack of social support, stressful life events, and social demographic variables (female gender) (25, 26).

Addressing the wellbeing, positive mental health, and mental disorders in older adults in an integrated manner remains a challenge. In this context, variables such as sociodemographic and economic status, perception of health satisfaction, family life, living arrangements, physical health, caregiver burden, and the role of social support may be significant. The aim of this study was to assess the relationship between life satisfaction, positive affect, and symptoms of depression and anxiety with sociodemographic, psychosocial, and clinical variables, also to identify the relative importance of these predictor groups.

## 2. Methods

### 2.1. Sample

This paper reports baseline results from a prospective cohort study conducted in Chile. The study included a probabilistic, polyethapic, cluster, and stratified sample of people between 60 and 80 years of age, living in two large urban areas of central-southern Chile. The areas comprised Gran Santiago, which encompasses the province of Santiago with 32 municipalities, including two other highly populated municipalities named Puente Alto and San Bernardo, as well as the Province of Concepción, with 12 municipalities. According to the last national census, these two metropolitan areas account for 40.5% of the national population (34.8% Gran Santiago and 5.7% Concepción), with up to 17.5 million inhabitants (1).

In Chile, provinces are subdivided into municipalities, followed by districts and blocks. In Santiago, the 34 municipalities were stratified in nine groups of communes based on the median income per capita, the proportion of multidimensional poverty, and the average level of education. Within each of these groups, the municipality, blocks, and housing units were considered as nested sampling units. All municipalities and blocks were randomly selected, except for one cluster in Santiago (comprising Vitacura, Providencia, and Las Condes). A high-income zone where a snowball strategy was employed. In Concepción, the commune was stratified, considering the block as the primary sampling units and the housing units within each block as the secondary sampling units. A proportional number of blocks based on the estimated population of older adults were sampled within each stratum. The blocks were randomly selected using spatial sampling, and census tracts with less than four individuals within the age range, as well as blocks with insufficient population (minimum of 30 or 50 people) based on the 2017 census, were excluded. Within each block, a sampling frame was created based on housing units, from which three main housing units and three replacement units were randomly selected. Each selected household was visited at least three times, and in cases where the interview was not successful, a replacement household was chosen. In each housing an older adult was randomly selected to be surveyed. Individuals who were transiently living in the selected household, homeless, or institutionalized were excluded.

To determine the necessary sample size, a linear multiple regression fixed model with a medium effect size of 0.15, a 95% level of significance and 50 predictor variables was considered. A power of 80% was achieved with 238 participants.

The surveys were conducted simultaneously at both sites between August and December 2021.

### 2.2. Measures

Predictive models were generated for the outcome variables: life satisfaction (SWLS) (27, 28), positive affect (PANAS) (29), depressive symptoms using PHQ-9 (30), and anxiety assessed using the geriatric anxiety inventory (GAI) (31). The psychometric properties of all the aforementioned instruments have been studied in the Chilean population and have shown positive indicators in different populations, including the elderly (32–35).

Considering the large number of predictor variables (36), the independent variables were organized into 16 groups using both theoretical and practical criteria: 1. Age; 2. Sex; 3. Other demographics; 4. Caregiver status; 5. Social participation (37); 6. Economics status; 7. Household members; 8. Medication use (38); 9. Alcohol use (38); 10. Chronic diseases; 11. Musculoskeletal disorders; 12. Cancer; 13. Sense organ diseases; 14. Other diseases (39, 40); 15. Satisfaction with health status; 16. Satisfaction with family life (Table 1).

**Table 1:** Group of variables in dominance analysis.

Group	Group name	Variables	Sample	
			Unweighted	Weighted^a^
1	Age	Age	*M* = 69.2	*M* = 69.2
2	Sex	Female	66.40%	57.40%
		Male	33.60%	42.60%
3	Other demographics	Geographical area		
		Concepción	62.40%	14.40%
		Gran Santiago	37.60%	85.60%
		Education level: (years)	*M* = 10.7	*M* = 11.3
		Marital status		
		Married-legal partner	50%	57.90%
		Widow	17.90%	12.30%
		Divorced	18.90%	20.20%
		Never married	13.10%	9.60%
4	Caregiver status	Yes	27%	38.50%
5	Social participation	Neighborhood council	26%	26.80%
		Sports club	22.20%	7.10%
		Religious group	27.50%	14.90%
		Artistic group	5.60%	9.40%
		Older adults’ group	11.90%	12.80%
		Volunteer group	3%	4.30%
		Self-help group	2.50%	2.90%
		Other	3.30%	4.80%
6	Economics status	Employment status		
		Not working	71.70%	71.80%
		Part-time	17.10%	13.80%
		Full-time	11.10%	14.40%
		Economic satisfaction*	*M* = 3.5	*M* = 3.5
7	Household members	Living alone	13.80%	9.50%
		Living with others: couple	51.80%	60.60%
		Parents	5.60%	7.00%
		Children	47.20%	50.60%
		Grandchildren	27%	30.70%
		Other relatives	11.90%	12.10%
		Other non-relatives	14.10%	11.60%
8	Medication use	No use	16.50%	18.90%
		Use: 1–2 medicaments	28.90%	21.40%
		3–5 medicaments	34.70%	40.70%
		6 or more	19.80%	18.80%
9	Alcohol use	No	61.40%	49.30%
		Yes: Almost all days	6.60%	10.40%
		3–4 times a week	3.80%	2.30%
		1–2 times a week	9.80%	12.90%
		1–3 times a month	11.60%	17.30%
		Less than 1 time a month	6.80%	7.84%
10	Chronic diseases	Diabetes	26.30%	26.50%
		Hypothyroidism	19.90%	19.20%
		Renal disease	5.05%	8.90%
		Hypertension	57.60%	58%
		Cardiac disease	11.90%	14.40%
		Peripheral vascular disease	11.40%	15.30%
		Cerebrovascular disease	4.30%	2.80%
11	Musculoskeletal disorders	Hip fracture	6.60%	6.60%
		Arthrosis	38.40%	35.40%
12	Cancer	Yes	3.50%	4.70%
13	Sense organ diseases	Low vision	73.20%	67.80%
		hearing impairment	27.70%	31%
14	Other diseases	Respiratory diseases	16.90%	14.50%
		Parkinson’s disease	0.80%	1.90%
		Hepatic diseases	2.30%	4.90%
15	Satisfaction with health	Satisfaction with health	*M* = 3.59	*M* = 3.45
16	Satisfaction with family life	Satisfaction with partner cohabitation*	*M* = 4.41	*M* = 4.45
		Satisfaction with home cohabitation*	*M* = 4.47	*M* = 4.38

^a^A weight was used to account for the probability of the municipality, block, household, and respondent being selected. A second weight was utilized to adjust the data to the 2017 national census on the basis of age, gender, and marital status.

*Five levels.

### 2.3. Procedure

The face-to-face interviews were conducted by trained interviewers with experience in interacting with older people. Interested professionals underwent an eight-hour training session, one at each site, led by different researchers according to their research area and professional competence. The trainees received instructions on the selection and contact of the study subjects. Approximately 80% of them were selected as interviewers and assigned blocks to visit. The visits were recorded in ad-hoc files, including the result of each contact. The interviews were carried out in each participant’s home and began with the reading and signing of an informed consent form, followed by the collection of contact information for follow-up. The interviewers received continuous supervision and feedback. Each interview was reviewed by a fieldwork coordinator, and any doubts were resolved by the interviewer, who sometimes had to make a second visit to the house for this purpose. Data monitoring was done in a digital file, which allowed for timely follow-up of the measurements in both study locations.

This study was approved by the Ethics, Bioethics, and Biosafety Committee of the University of Concepción, and each participant expressed their willingness to participate in the study by signing an informed consent form. The data were anonymized for entry into the database, and procedures were established to refer eventual suspected clinical and/or psychosocial cases to the concerned healthcare facility.

### 2.4. Data analyses

The surveys, recorded on paper, were independently entered into Excel spreadsheets by two data entry operators, with manual correction of any differences to obtain a preliminary analysis database. Subsequently, the logical consistency of the data was analyzed to eliminate errors. Multiple imputations were performed using fully conditional specification, with 20 imputed databases and 30 iterations. Considering the complex sampling structure, all the analyses were conducted based on the final analysis database according to sampling design stratification by municipalities or groups of municipalities, as well as the primary and secondary sampling units for each stratum. Each case was initially weighted by the size of the stratum and the probability of choice of each sampling unit. Subsequently, sample was post-stratified using the 2017 census data, considering the population size by gender and decade.

First, a descriptive analysis of the anxious and depressive symptoms, life satisfaction, and PA was conducted by geographical area and for the total sample, considering the relative size of each population. For anxiety and depressive symptoms and life satisfaction, the results were interpreted based on the cut-off scores and categories available in the literature.

Second, the relationship between sociodemographic and clinical variables with the criterion variables was analyzed using regression analysis for complex sample designs. For each criterion variable, a model was adjusted based on all the predictor variables (43, organized into 16 groups) to analyze the coefficients associated with the pertinent variables within the groups that appeared significant.

Finally, a dominance analysis of the predictor groups was performed to estimate the relative significance of each one, without the limitations presented by standardized beta values or partial/semi-partial correlations (41). The relative importance of a predictor with respect to another was defined as the difference in explicative power, measured by a fit-index like R^2^, between models that included the first predictor vs those that included the other. Three types of dominance were described: complete dominance, conditional dominance, and general dominance. A predictor, X, completely dominates another, Y, when the relative importance of X is higher than that of Y on each possible submodel that includes all other predictors. Conditional dominance is defined over the mean of all submodels of the same number of predictors. General dominance was evaluated using the average of all submodel means for each variable, which corresponds in linear models to the square semi-partial correlation and is also known as Shapley Value (42).

All these analyses were performed in R 4.2 using the *mice*, *dominance analysis*, and *survey* packages.

## 3. Results

### 3.1. Participant characteristics

A total of 396 participants responded to the questionnaires: 247 from the province of Concepción and 149 from Gran Santiago. Table 2 shows that the sample had a higher proportion of women (66%). The sample is balanced between younger older adults, between 60 and 69 years (52%) vs the still older ones between 70 and 80 years (48%). Among the older adults, 50% were married, 49% had incomplete basic or secondary education, while 72% were unemployed.

**Table 2:** Sociodemographic characteristics of the sample (*N* = 396).

		Concepción		Gran Santiago		Total	
		N	%	N	%	N	%
Gender	Male	86	35	47	31	133	34
	Female	161	65	102	69	263	66
Age	60–64	60	24	44	29	104	26
	65–69	68	27	36	24	104	26
	70–74	66	27	24	16	90	23
	75–80	53	22	45	30	98	25
Marital status	Married	120	48	78	52	198	50
	Widower	52	21	19	13	71	18
	Divorced	39	16	36	24	75	19
	Never married	36	15	16	18	52	13
Studies	Basic education or incomplete secondary education	134	54	58	40	192	49
	Complete secondary education	51	21	32	22	83	21
	Higher education	61	25	54	38	115	29
Employment situation	Full time work	23	9	21	14	44	11
	Part time work	44	18	24	16	68	17
	No work	180	73	104	70	284	72

In analyzing the mental health variables (see Table 3), we can observe a significant difference in the levels of depressive symptomatology by geographical area: a slight to moderate difference, with higher levels in Gran Santiago compared to those in Concepción. In the rest of the indicators, small, non-significant differences were observed.

**Table 3:** Life satisfaction, positive affect, depressive symptoms, and anxiety symptoms, in total and by geographic area.

	Total		Concepción		Gran Santiago		*t*-test^a^	*d*
	*M*	*SD*	*M*	*SD*	*M*	*SD*		
Life satisfaction (SWLS)	27.81	6.29	27.14	6.1	27.93	6.33	*t*(8) = 0.963, *p* = 0.364	0.12
Positive affect (PANAS)	37.64	9.27	38.95	7.79	37.42	9.5	*t*(8) = −1.610, *p* = 0.146	0.16
Depressive symptoms (PHQ-9)	7.11	6.2	4.8	5.07	7.5	6.3	*t*(8) = 4.148, *p* = 0.003	0.43
Anxiety symptoms (GAI)	7.59	5.74	7.08	5.93	7.67	5.71	*t*(8) = 0.950, *p* = 0.370	0.1

^a^*t*-test adapted to a complex sample of difference between regions.

In analyzing life satisfaction according to the categories proposed by the authors of the SWLS (43), the most frequent categories were very satisfied (38.4%) and satisfied (36%), and in the case of PA, most of them had very high (46.7%) and high (32.7%) levels of affect.

Table 4 presents the responses to the PHQ-9 are. Considering the participants above the cut-off point of 10 points, which indicating risk of depression (30), there was a higher proportion of cases with higher levels of symptomatology in Gran Santiago compared to Concepción. The moderate category had a 1.56 times higher proportion, the moderate to severe category had a 2.4 times higher proportion, and the severe cases had a 3.3 times higher proportion in Gran Santiago. Regarding anxiety symptomatology, 31.6 and 21.6% of the population had a GAI score equal to or higher than 11 and 13 respectively. Both cut-off scores indicate a risk to anxiety disorder (36).

**Table 4:** Distribution of PHQ-9 scores by region and in total.

Region	Minimum (0–4)	Mild (5–9)	Moderate (10–14)	Moderate to severe (15–19)	Severe (20–27)
Concepción	61.27	20.19	13.06	3.94	1.55
Gran Santiago	35.23	29.84	20,35	9.49	5.08
Total	38.98	28.45	19.3	8.69	4.57

### 3.2. Relationship between outcomes and sociodemographic, psychosocial, and clinical variables

For each criterion variable, a model that included all 16 groups of predictor variables was tested. This model was statistically significant for all criterion variables: life satisfaction, *F*(52, 793697.7) = 76.852, *p* < 0.001, $R^{2}$ = 0.55; positive affect, *F*(52, 596935.8) = 30.306, *p* < 0.001, $R^{2}$ = 0.52; depressive symptomatology, *F*(52,110594.4) = 119.418, *p* < 0.001, $R^{2}$ = 0.64; and anxiety symptomatology, *F*(52, 143814.8) = 79.22, *p* < 0.001, $R^{2}\;$ = 0.52. Therefore, for each criterion variable, we analyzed which groups of predictors were significant, as well as the relative importance of each of them, using dominance analysis as presented in Table 5.

**Table 5:** General dominance and significance of set of predictors.

Set of predictors	PHQ9		GAI		Life satisfaction		Positive affect	
	GD	*p*	GD	*p*	GD	*p*	GD	*p*
Age	0.001	0.907	0.001	0.710	0.010	0.494	0.010	0.004
Sex	0.029	<0.001	0.007	0.057	0.004	0.982	0.014	0.038
Demographics	0.033	<0.001	0.063	<0.001	0.035	0.017	0.012	0.042
Caregiver status	0.002	0.111	0.008	0.861	0.019	0.043	0.004	0.545
Social participation	0.040	0.011	0.020	0.201	0.082	<0.001	0.038	<0.001
Economics status	0.086	<0.001	0.065	<0.001	0.040	<0.001	0.059	<0.001
Household members	0.044	0.001	0.030	0.501	0.080	0.013	0.104	<0.001
Medication use	0.051	0.016	0.039	0.141	0.012	0.501	0.025	0.277
Alcohol use	0.029	0.016	0.012	0.678	0.099	<0.001	0.037	<0.001
Chronic diseases	0.064	0.005	0.085	0.017	0.032	<0.001	0.056	<0.001
Musculoskeletal disorders	0.019	0.118	0.007	0.422	0.033	0.021	0.013	0.008
Cancer	0.006	0.919	0.003	0.297	0.017	0.030	0.006	0.696
Sense organ diseases	0.030	0.398	0.011	0.500	0.012	0.913	0.010	<0.001
Other diseases	0.032	0.007	0.020	0.052	0.030	0.009	0.010	0.657
Satisfaction with health	0.149	<0.001	0.141	<0.001	0.012	0.270	0.109	<0.001
Satisfaction with family life	0.020	<0.001	0.008	0.418	0.031	<0.001	0.008	<0.001
Total^a^	0.637	<0.001	0.521	<0.001	0.547	<0.001	0.516	<0.001

The significance of each predictor group is evaluated using hierarchical regression, comparing the model with all the variables and another that omits the variables assigned to the group. GD, General dominance, using R^2^ adapted to complex-sample.

^a^Significance is evaluated between the full model and the null model (without predictors) and GD correspond to R^2^ for the full model.

In the case of life satisfaction, the overall model was significant: *F*(52, 793697.7) = 76.852, *p* < 0.001, $R^{2}$ = 0.55. Eleven groups of significant predictors were observed and ordered according to their general dominance: alcohol use, social participation, household members, economic variables, other demographics, musculoskeletal disorders, chronic disorders, satisfaction with family life, other diseases, caregiver status, and cancer. Although alcohol use conditionally dominated all the other predictors, in terms of complete dominance, it was equal in importance to social participation, both dominating 10 other groups of predictors. When studying the coefficients specific to alcohol use, it is of interest that, compared to not consuming alcohol, the coefficient for 1 to 4 times a month (*β* = 2.007, *p* = 0.003) is significant and positive. However, the coefficient for almost every day (*β* = −4.804, *p* < 0.001) was negative, which shows that casual alcohol consumption was associated with greater life satisfaction than daily consumption. A lower life satisfaction was associated with caregiver status (*β* = −1.612, *p* = 0.043), belonging to an artistic (*β* = −3.60, *p* = 0.001) or self-help association (*β* = −6.62, *p* < 0.001), having a hip fracture (*β* = −3.006, *p* = 0.006), Parkinson’s (*β* = −4.69, *p* = 0.023), and cancer (*β* = −3.70, *p* = 0.03). Living in Gran Santiago rather than living in Concepción (*β* = 2.134, *p* = 0.002), greater satisfaction with economic needs (*β* = 1.724, *p* < 0.001), greater satisfaction with home cohabitation (*β* = 1.519, *p* < 0.001), living alone (*β* = 4.41, *p* = 0.005), presenting cardiac disease (*β* = 3.824, *p* < 0.001), and presenting liver disease (*β* = 4.37, *p* = 0.047) were associated with greater life satisfaction.

It is generally expected that diseases will have a negative effect on life satisfaction. Therefore, a possible suppressing effect was evaluated by reviewing the regression coefficients of the models that separately consider each variable. The effects of liver disease (*β*_0_ = 6.152, *p* < 0.001) was positive and statistically significant, even when no other variables were included in the model, so a suppression effect is missing, although the low frequency of these cases (*n* = 9) should be considered. The effect of cardiac disease was not statistically significant (*β*_0_ = 1.26, *p* = 0.217), which confirms the suppression effect for this variable.

In the case of PA, the general model was also significant: *F*(52, 596935.8) = 30.306, *p* < 0.001, $R^{2}$ = 0.52. Twelve significant predictors were observed and ordered according to their overall dominance: satisfaction with health, household members, economic variables, chronic diseases, social participation, alcohol use, sex, musculoskeletal disorder, other demographics, sense organ diseases, age, and satisfaction with family life. Considering conditional and complete dominance, household members and health satisfaction stood out as they both conditionally dominated 14 other groups of predictors, while household members completely dominated 12 groups and health satisfaction dominated the remaining 11 groups. Lower PA was associated with older age (*β* = −0.231, *p* = 0.004), living with children (*β* = −6.037, *p* < 0.001), and higher PA associated with being male (*β* = 2.397, *p* = 0.038), working part-time compared to not working (*β* = 4.672, *p* < 0.001), having hypothyroidism (*β* = 3.321, *p* = 0.001) and vascular problems (*β* = 3.312, *p* = 0.018), osteoarthritis (*β* = 3.127, *p* = 0.002), low vision (*β* = 3.407, *p* < 0.001), greater satisfaction with health (*β* = 4.309, *p* < 0.001), and greater satisfaction with home cohabitation (*β* = 1.589, *p* < 0.001). Regarding social participation, participating in religious associations (*β* = 2.366, *p* = 0.021) and participating in senior citizen groups (*β* = 3.5, *p* < 0.001) were associated with greater PA, while participating in sports (*β* = −4.45, *p* = 0.001) and artistic clubs (*β* = −3.508, *p* = 0.022) were associated with less PA. When considering alcohol use, drinking one to three times a month was associated with lower PA than not drinking (*β* = −3.76, *p* = 0.006). However, drinking one to two times a week was associated with higher PA than not drinking (*β* = 2.55, *p* = 0.021). A potential suppressing effect, similar to life satisfaction, was evaluated by reviewing the regression coefficients of the models that separately consider each variable. The analysis confirms the suppression effect since lower value coefficients and non-statistically significant coefficients were observed in all variables: hypothyroidism (*β*_0_ = 1.498, *p* = 0.584), vascular problems (*β*_0_ = 1.597, *p* = 0.279), osteoarthritis (*β*_0_ = 0.079, *p* = 0.970), and low vision (*β*_0_=−0.649, *p* = 0.736).

In the case of depressive symptoms, the model that considers all the variables was significant: *F*(52,110594.6) = 119.418, *p* < 0.001, $R^{2}$ = 0.64. When analyzing by groups of predictors, 11 of them were significant, ordered by their general dominance: satisfaction with health, economic variables, chronic disorders, medication use, household members, social participation, other demographics, other diseases, alcohol use, sex, and satisfaction with family life. When performing a complete dominance analysis, satisfaction with health stands out, dominating all other groups, except for economics variables. Although the economic variables that follow in importance do not completely dominate the rest of the groups, they do so conditionally when considering models with an equal number of predictor groups. When analyzing the specific coefficients, the following variables were associated with lower depressive symptomatology: being male compared to being female (*β* = −2.56, *p* < 0.001), participation in associations for the elderly (*β* = −2.07, *p* = 0.006), other institutions (*β* = −2.66, *p* = 0.044), greater satisfaction of economic needs (*β* = −1.59, *p* < 0.001), working part-time compared to not working (*β* = −2.91, *p* < 0.001), drinking alcohol less than once a month compared to not drinking alcohol (*β* = −2.54, *p* = 0.008), greater satisfaction with health (*β* = −2.72, *p* < 0.001), and greater satisfaction with home cohabitation (*β* = −1.25, *p* < 0.001). Greater depressive symptomatology was associated with living in Gran Santiago compared to living in Concepción (*β* = 1.82, *p* < 0.001), being widowed compared to being married (*β* = 3.18, *p* = 0.032), living with grandchildren (*β* = 1.56, *p* = 0.038), and presenting a vascular (*β* = 2.27, *p* = 0.018) or respiratory disorder (*β* = 1.57, *p* = 0.012). A higher amount of medication intake was associated with a higher level of depression, specifically taking 1–2 (*β* = 2.32, *p* = 0.005) and 4 or more (*β* = 2.89, *p* = 0.004) compared to not taking medication.

Finally, in the case of anxiety symptomatology, the model that considers all variables was significant: *F*(52, 143814.8) = 79.220, *p* < 0.001, $R^{2}\;$ = 0.52. A smaller number of groups of significant predictors were observed, four in total, ordered according to their general dominance: satisfaction with health, chronic disorders, economic variables, and other demographics. Satisfaction with health conditionally dominated all the other groups of predictors, although not completely, since chronic disorders dominate in some specific models. When analyzing the coefficients of the significant groups, it was observed that greater satisfaction with health (*β* = −2.57, *p* < 0.001), greater number of years of education (*β* = −0.339, *p* < 0.001), greater satisfaction of economic needs (*β* = −1.27, *p* = 0.001), and working part-time compared to not working (*β* = −1.66, *p* = 0.005) presented negative relationships with levels of anxiety symptomatology. With respect to clinical factors, developing renal (*β* = 3.58, *p* = 0.023) or vascular (*β* = 1.67, *p* = 0.035) disease increased the levels of anxiety symptomatology.

**Figure 1:**
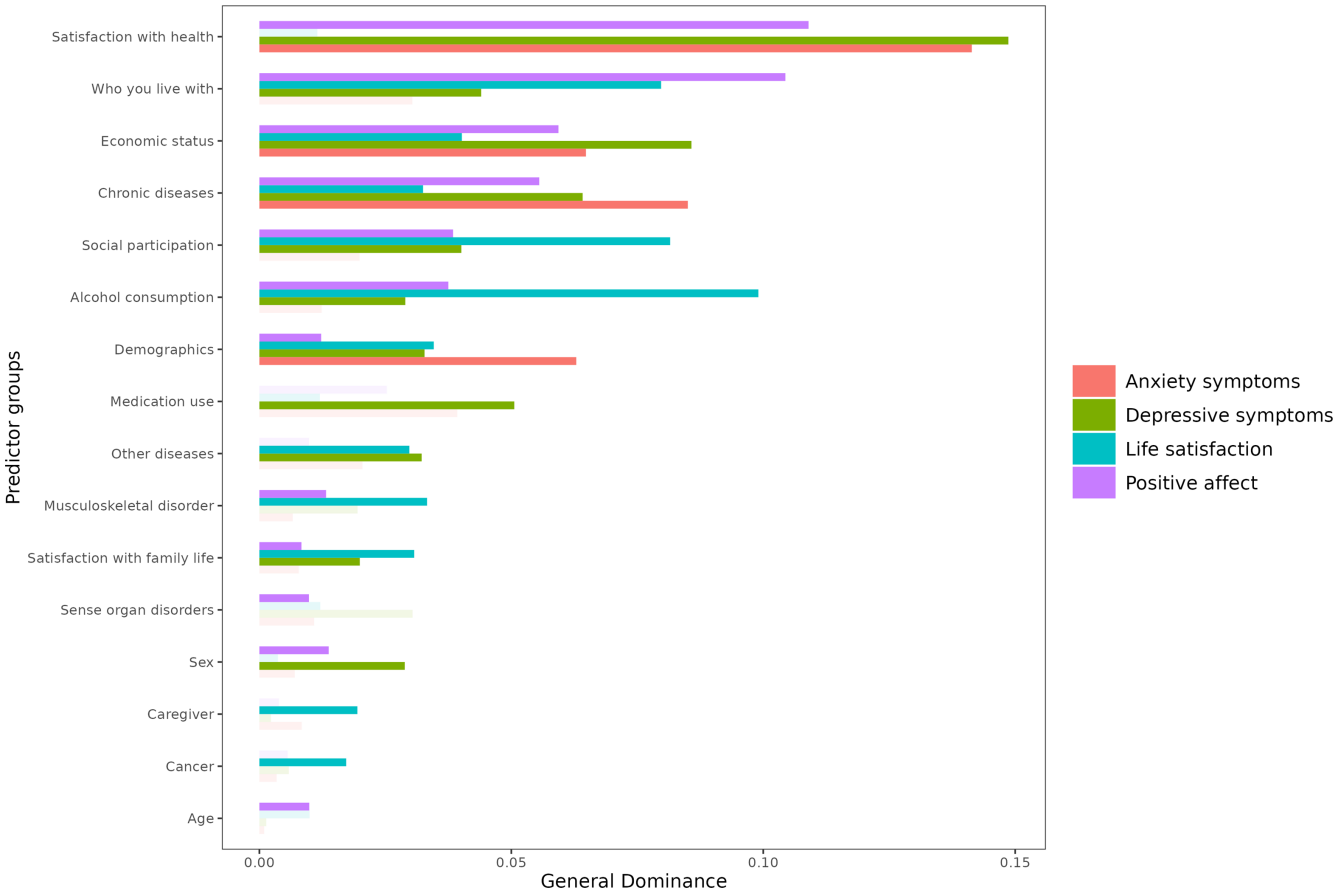
General dominance of predictor groups for outcome variables.

Figure 1 shows a summary of the results of the general dominance analysis for the outcome variables. The results of the complete regression models are available as Supplementary material on https://osf.io/t9w8b/?view_only=9b37aa3dde4b48b8b2fa7624d6a745d9.

## 4. Discussion

The aim of this study was to analyze the behavior of two indicators of personal wellbeing (life satisfaction and positive affect) and two mental health issues (depressive and anxiety symptomatology) in a random sample of the elderly. The results confirm the wide heterogeneity that is present in the elderly population, contrary to common stereotypes (44). The reported level of life satisfaction in the sample was very high in almost 40% of the participants and high in a further third. In relation to the presence of positive affect, almost half of the participants reported a very high level of positive affect and just over a further third, a high level. Around 20% reported low or very low levels. In relation to the indicators of mental health issues, one-fifth of the participants reported high or severe levels of depressive symptomatology, and a further quarter reported moderate levels. One in five participants was at an increased risk of being diagnosed with an anxiety disorder. Taken together, it can be concluded that the majority of the older adult population showed positive indicators of personal wellbeing and mental health.

Overall, the results show a partial overlap of predictors for each of the conditions studied. The most significant difference was observed between the predictors of the assessed conditions that were affective (positive affect, depressive and anxious symptomatology) and the predictors of life satisfaction, a variable that alludes to an evaluative process about one’s own life.

Regarding the composition of the groups of significant predictors found for two indicators of positive mental health, for the case of life satisfaction, eight groups (social participation, household members, economic variables, other demographics, musculoskeletal disorders, chronic diseases, other diseases, and satisfaction with family life) are in line with results from previous studies exploring factors affective life satisfaction in older adults (6, 7). However, alcohol use, caregiver status and cancer were not present in these studies. Of these three groups, the last two were the least important in terms of general dominance, however, alcohol use conditionally dominated all other predictors, in terms of complete dominance. This result is expanded further below. For the case of PA, we found twelve significant groups of predictors (satisfaction with health, household members, economic variables, chronic diseases, social participation, alcohol use, sex, musculoskeletal disorder, other demographics, sense organ diseases, age, and satisfaction with family life), these groups show a limited overlap with the predictors of life satisfaction; however, comparing with previous studies, results are mixed, there is only similarities with covariables such as marital status, social participation, socioeconomic status, emotional health, education and sex, but not with cognitive functioning, ethnicity, health behaviors and physical activity, which are all variables not addressed in this study (10).

The composition of the groups of significant predictors found for two indicators of mental health predictors was different, with depression showing eleven significant groups of predictors (satisfaction with health, economic variables, chronic disorders, medication use, household members, social participation, other demographics, other diseases, alcohol use, sex, and satisfaction with family life) and anxiety with only four significant groups of predictors (satisfaction with health, chronic disorders, economic variables, and other demographics), but with all showing a total overlap with depression. Our results show similarities with previous studies were sex, alcohol use, economic variables, living arrangement, presence of physical disorders chronic diseases, poor vision, and hearing loss are considered risk factors for depression and anxiety in the elderly (16, 20, 25).

A wide range of variables were included to identify the dominant predictors of these measures of wellbeing and mental health problems.

Satisfaction with health was the dominant predictor for depression, anxiety, and positive affect, but not regarding life satisfaction. With respect to anxiety, satisfaction with health and the presence of chronic disease were both ranked as the second dominant predictor, which was not found in the case of depression and positive affect. In the case of depression, in addition to satisfaction with health, economic wellbeing was the second most dominant predictor, for positive affect, it was household membership. These results confirm that perceived health, rather than the “objective” health condition, is ultimately the closest determinant of emotional wellbeing (45–47), and opens questions about the eventual mediating role it may play between the presence of physical illness and positive mental health (48).

Along with social participation and household membership, alcohol use was the predictor that showed dominance in relation to life satisfaction. Although alcohol use was also associated with depression and positive affect, its relationship with these conditions was not as strong as that with life satisfaction. A detailed analysis of the relationship between alcohol use and the conditions studied showed the importance of paying attention to the frequency of alcohol use. Daily drinking, which was present in 10% of the participants, was negatively related to life satisfaction, reflecting the risks of alcohol abuse (49, 50). In contrast, occasional drinking was associated with higher positive affect and lower depressive symptomatology, an unexpected result that could be related to a more active social life. Such results would require further exploration in future studies.

A predictor that showed strong relationships with all the conditions studied was satisfaction with personal economic situation. This indicated the weight that perceived economic security has on the wellbeing of the population studied. Similarly, satisfaction with family life had high dominance over positive affect, but it also weighs on life satisfaction and the presence of depressive symptoms. This shows the important role that the network of close relationships plays in this population. Similarly, social participation plays a relevant role in life satisfaction, positive affect, and the presence of depressive symptoms, highlighting the role that peer relationships play at this stage of life.

No effects related to the sex of the participants were observed in the conditions studied, except in relation to depressive symptomatology, which was more associated with women, and higher PA associated with being male. In depression symptoms, the relative importance of sex was weaker compared to nine other predictor groups. While the relationship between the female sex and greater depressive symptomatology was well established, attenuation of this difference has been observed in the older adult population (51).

We have discussed the predictors that showed a clearer and more intense pattern through the dominance analysis with respect to the assessed conditions. However, some additional predictors are of particular interest for further research. The higher frequency of depressive symptomatology in Gran Santiago compared to that in Concepción may be indicative of the relationship between urban life and its characteristics as well as people’s wellbeing and mental health. Although this study does not allow us to identify what could explain this greater symptomatology in the more populous city, it invites us to explore this aspect. The age of the participants was also not a predictor of the conditions studied, except in relation to positive affect, which was lower in participants over 70 years of age. A lower emotional intensity with increasing age is a hypothesis that has been put forward by several researchers (47, 52). Finally, it is interesting that working part-time was shown to be related to less depressive and anxiety symptomatology, although it was also related to less positive affect.

It is noteworthy that half of the study participants reported living with one of their children, and almost a third with their grandchildren. However, neither of these conditions was positively related to the variables studied. Instead, living with children was associated with lower positive affect, and living with grandchildren with greater depressive symptomatology. This suggests the need for developing studies to clarify the contexts in which living with children and grandchildren is beneficial for older adults and those in which it could be negative for their wellbeing.

This study assessed four components of positive and negative mental health of the elderly in a random sample of the two largest population areas in Chile, as well as a large number of predictor variables that allowed us to detect previously unknown associations. However, the study does have limitations. The main one is the cross-sectional nature of the data, which does not allow for establishment of the directionality of the associations found. A challenge for future research is to find out what happens to older individuals with poorer mental health and lower wellbeing compared to those with positive mental health indicators. This is important given that in our sample, the most marked difference between predictors seems to be related to *affective* variables, such as positive affect and symptoms of anxiety and depression, against variables mediated by a *cognitive* assessment, for example, life satisfaction. A further exploration of this perspective may provide evidence on whether wellbeing, considered an indicator of positive mental health, and affective symptoms, which suggest negative mental health, represent divergent points on a continuum or traverse as different dimensions. Finally, we observed a positive association between some physical conditions and life satisfaction and PA. In some diseases, the number of cases is low (as liver disease), which questions the result. However, the relationship between some problems, like cardiac problems, and life satisfaction needs to be further explored by analyzing the role of economic needs and the degree of their satisfaction.

Simultaneously studying the positive and negative aspects of wellbeing and mental health of older adults allows for a more comprehensive view of the challenges at this stage of life. This study contributes to this perspective by identifying common and specific predictors of both dimensions. Exploring this aspect is one of the challenges for future research on the topic, with significant implications for the quality of life of older adults.

## Data availability statement

The raw data supporting the conclusions of this article will be made available by the authors, without undue reservation.

## Ethics statement

The studies involving human participants were reviewed and approved by the Ethics, Bioethics and Biosafety Committee of the University of Concepción. The patients/participants provided their written informed consent to participate in this study.

## Author contributions

SS designed the study, supervised collection of data, and wrote the manuscript. JA supported data analyses and co-authored the manuscript. FC co-designed the study, co-authored the manuscript, and critically reviewed it. CB co-designed the study and analyzed the data. CI supported the study design and data collection process. AC-C coordinated the data collection process and supported the manuscript review. All authors contributed to the article and approved the submitted version.

## Funding

This study was funded by Chilean National Agency for Research and Development, ANID (Project FONDECYT 1201158 to SS). FONDECYT had no role in the design of the study, collection, analysis, interpretation of data, and in writing the manuscript.

## Conflict of interest

The authors declare that the research was conducted in the absence of any commercial or financial relationships that could be construed as a potential conflict of interest.

## Publisher’s note

All claims expressed in this article are solely those of the authors and do not necessarily represent those of their affiliated organizations, or those of the publisher, the editors and the reviewers. Any product that may be evaluated in this article, or claim that may be made by its manufacturer, is not guaranteed or endorsed by the publisher.
